# Using Communication Tools to Explore Young Siblings’ Experiences of Having a Brother or Sister with Pediatric Palliative Care Needs

**DOI:** 10.3390/children9050641

**Published:** 2022-04-29

**Authors:** Ulrika Kreicbergs, Stefan Nilsson, Margaretha Jenholt Nolbris, Malin Lövgren

**Affiliations:** 1 Department of Caring Sciences, Palliative Research Center, Marie Cederschiöld University, P.O. Box 11189, 100 61 Stockholm, Sweden; malin.lovgren@esh.se; 2 Department of Women and Children’s Health, Karolinska Institutet, 171 77 Stockholm, Sweden; 3 Institute of Health and Care Sciences, University of Gothenburg Centre for Person-Centered Care, Sahlgrenska Academy, P.O. Box 457, 405 30 Gothenburg, Sweden; stefan.nilsson.4@gu.se (S.N.); margaretha.nolbris@fhs.gu.se (M.J.N.); 4 The Queen Silvia Hospital, Sahlgrenska University Hospital, Child Medicine, 416 50 Gothenburg, Sweden; 5 Advanced Pediatric Home Care, Astrid Lindgren Children’s Hospital, Karolinska University Hospital, 171 76 Stockholm, Sweden

**Keywords:** siblings, bereavement, palliative care, communication, emotions

## Abstract

Siblings of children with palliative care needs often suffer feelings of being neglected, and their needs for information and involvement are frequently unmet. This study aims to explore the experiences and feelings of siblings of children with palliative care needs, and to determine what is important to them. Nine siblings, aged 6–14 years, were interviewed using four different communication tools: See-Hear-Do pictures, including the empty body as a separate element, Bear cards, and words originating from previous sibling research. Data were analyzed using conventional content analysis. Five categories emerged concerning aspects that the siblings described about their situation and things that they found important: being part of a special family; school—a place for leisure, friends, and learning; relentless feelings of guilt and self-blame; losses and separations; and awareness of death—not if, but when. Siblings of children with rare diseases expressed an awareness that their brother or sister would die, although still felt they were part of a special, happy family. Siblings of children with palliative care needs due to an accident described relentless feelings of self-blame and guilt. The needs of siblings may vary depending on the condition that resulted in the ill sibling’s palliative care needs.

## 1. Introduction

Living with severe illness in the family is known to be stressful for all family members, and being a sibling living in the shadow of a severely ill brother or sister is no exception. Previous research has found that these siblings differ significantly from their peers in the general population, both socially and in terms of their quality of life [1]. Much of the sibling research has been performed in a pediatric oncology context, while the perspectives of those living with a brother or sister with another disease or disability are relatively unexplored. In a review of experiences among siblings with a chronically ill brother or sister, only 6 of 28 studies were based on siblings of a brother or sister with an illness other than cancer. It was found that siblings experienced negative psychological and emotional symptoms and post-traumatic stress, and that these symptoms interfered with their school functioning [2]. Problems in school have also been reported by Malkolm and co-workers, who studied siblings of children with rare life-limiting conditions and found that bullying was a problem they experienced. Two of the eight siblings interviewed reported that they had been teased by peers in school because of their ill brother’s or sister’s condition, and that teachers or other adults had not intervened. This may reflect a social attitude towards disabilities in society [3].

Winger, Kvarme, Løyland, Kristiansen, Helseth, and Ravn [4] examined families’ experiences of pediatric palliative home care; in 3 of the 23 articles reviewed the sibling’s voice was heard while in others it was their parents, mainly the mothers, who gave reports. It was found that families wanted respite care in order to be able to maintain a normal life. The authors stressed the need to include siblings’ perspectives in future studies. Family communication and family cohesion are known protective factors for psychosocial distress in families who have a child with severe illness or who has died [5,6]. Conversely, poor communication and lack of family cohesion are known risk factors for long-term psychosocial distress [7,8]. Jaaniste and co-workers recently published a paper focusing on parent–sibling communication. Thirty families with a severely ill child participated; 28 mothers, 2 fathers, and 46 siblings were studied as dyads and close to half of the siblings never or rarely initiated a conversation about their brother’s or sister’s illness or death [9]. Lack of information about the brother’s or sister’s prognosis can lead to siblings being absent at the time of death and can result in later regrets and ineffective grieving [7,10]. Siblings’ grief has been found to take considerable time. It has been reported that even two to nine years after the loss, most siblings report unfinished grief [11]. Cancer-bereaved siblings also describe lower self-esteem and maturity as compared with non-bereaved peers. A negative impact on schooling has also been reported among bereaved siblings together with poor adult socioeconomic outcomes [12]. Even higher mortality rates have been noted [13].

Little is known about the situations of siblings of a brother or sister with palliative care needs for a severe illness other than cancer. The aim of this study was, therefore, to use communication tools to explore their experiences and feelings, and further understand what is important to them in their situation. The study results may help healthcare professionals and families tailor the support given to siblings of children with palliative care needs and act accordingly.

## 2. Materials and Methods

### 2.1. Design and Population

This interview study involved siblings of children in need of round-the-clock palliative care services. Each participant took part in an individual semi-structured interview where they were asked to describe their experiences and feelings by using various communication tools. Inclusion criteria were that the siblings were non-bereaved, could speak Swedish, were aged 5–18 years, and had a brother or sister who was receiving or had received respite care at Sweden’s hospice for children and young people for a diagnosis other than cancer. The siblings were identified by the sibling supporter at the hospice who then informed the researcher so that they could contact the parents to invite the sibling to participate. Both parents and siblings received age-adapted written information about the aim of the study and the structure of the interviews. Nine siblings were willing to participate in interviews, which were conducted in their homes or at the researchers’ workplace during April to November 2019 (Table 1).

Written informed consent was obtained from the parents if the sibling was younger than 15 years, which was the case for all the siblings involved in this study. The study was conducted in accordance with the guidelines of the Declaration of Helsinki and approved by the Regional Ethical Review Board, Dnr. 1091-17, 19 April 2018.

### 2.2. Data Collection

The first and last authors conducted the interviews with the children. The interviews followed a guide that included four communication tools in order to build a dialogue about the siblings’ experiences and life situation. Four user-friendly, non-computer-based tools were used to ensure suitable options for siblings of all ages. The See-Hear-Do pictures developed for and often used in Swedish pediatric oncology were the first choice. In order to capture the emotional aspects more fully, Bear cards were used, along with 27 words often used in research to describe siblings’ situations. The blank body from the See-Hear-Do pictures was used so that siblings could draw and describe certain aspects of their situation. The siblings were asked to think about what it is like to be a sibling of a child with palliative care needs and what is important for them in their own life. Follow-up questions were asked throughout the interviews using the different communication tools so we could deepen our understanding, for example, “would you like to tell us more about these pictures/cards/words”?

**Figure 1:**
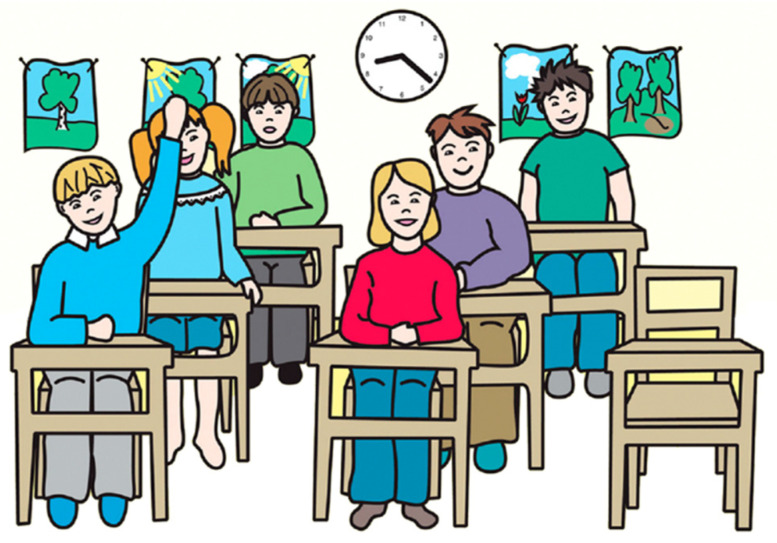
An illustration from among the See-Hear-Do pictures [14].

**Figure 2:**
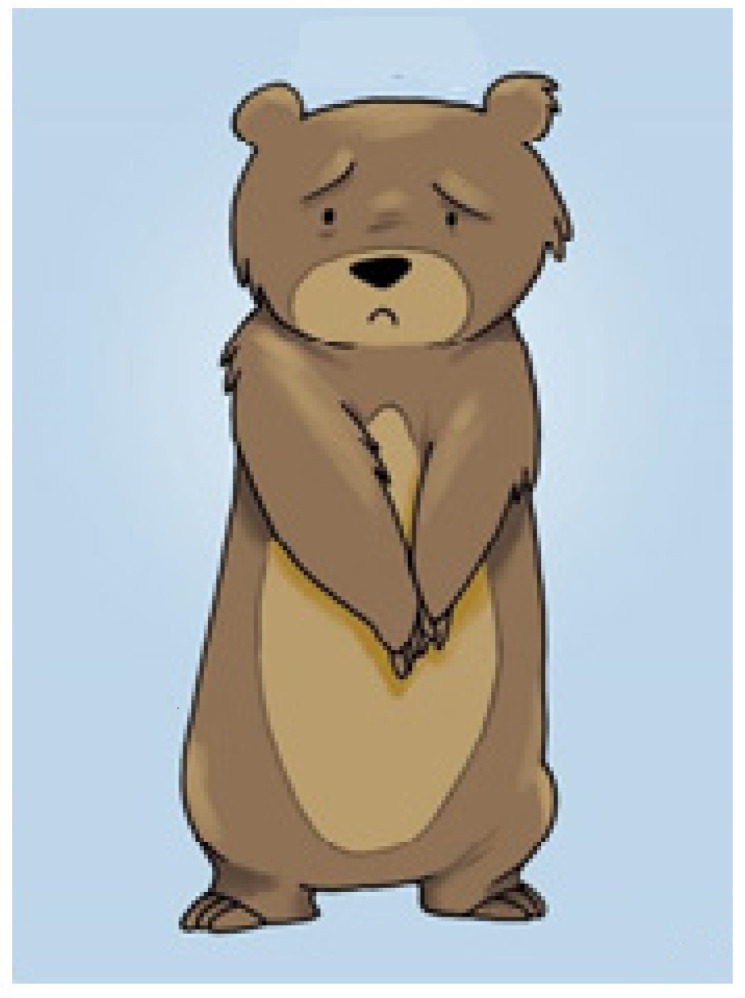
An example of a Bear card [15].

**Figure 3:**
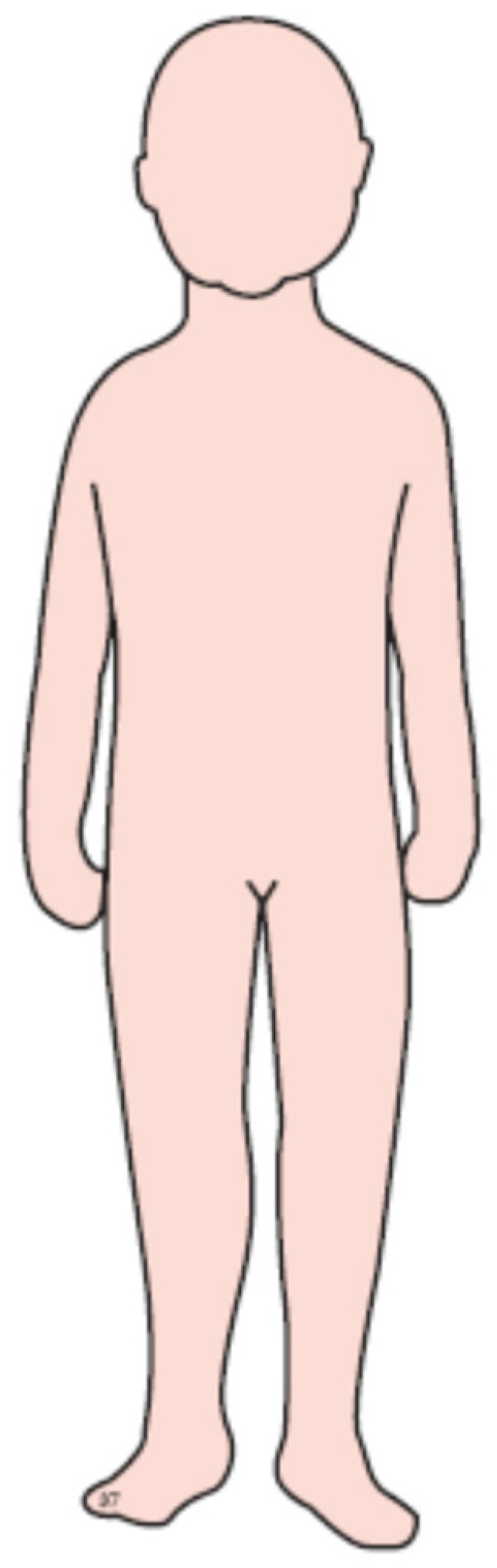
The blank body outline [14].

The following communication tools were used in the following order:

1. Each sibling selected an unlimited number of pictures from a selection of See-Hear-Do pictures [14]; these are part of a pedagogical teaching tool for children with cancer. The pictures illustrate illness-related subjects, treatment, and the daily environment around the child (Figure 1). The siblings were asked to choose pictures to describe what they value in their life. These pictures have been developed by healthcare professionals in pediatric oncology in Sweden and have been used for many years when talking to siblings in that context.

2. The siblings selected an unlimited number of images from the Bear cards. This tool was developed in Australia and consists of cards illustrating different emotions, e.g., anger, sadness, happiness, etc. [15] (Figure 2). The Bear cards were used to describe the siblings’ feelings about being a sibling of a child with palliative care needs or about any special event in their life. They were asked to choose images that either mirrored how they usually feel or help them talk about a particular moment; this was up to the sibling.

3. In the next step, from a range of words the siblings were asked to choose the words that described their experiences of being a sibling of a brother or sister with palliative care needs. The number of words they could choose was not fixed and the siblings were asked to talk more about the words chosen. The range of words presented was derived from previous research about siblings and their experiences of living with an ill brother or sister [16,17,18].
4. Lastly, the siblings were asked to draw and describe how they usually feel using a blank body outline (Figure 3).

After this, we asked if there was anything else that the siblings wanted to tell us.

All interviews were audio-recorded and transcribed verbatim. The interviews each took around 30 min (16–48 min) and were most often conducted with the sibling on their own. One or both parents were present at two interviews; in one case this was the choice of the sibling and in the other of the parent.

### 2.3. Data Analysis

Data were analyzed using conventional content analysis in accordance with Hsieh and Shannon [19]. Conventional content analysis is a method often used when there is a shortage of theories and literature concerning the studied phenomenon. Analysis began with a naïve reading of the transcribed interviews by all authors to gain a sense of the entirety of the data. Thereafter, meaning units were selected and sorted into codes based on similarities and differences in areas of special meaning that emerged from the different tools used in the sibling’s stories (first and last author). This work was data-driven rather than based on preconceptions and was conducted by the two authors independently. Thereafter, the codes were grouped into categories (first and last author) based on similarities in content. Examples of codes included the importance of school, friends, family, pets, medication, care, caring, daily life, feelings (good and bad), fear of change, relations, and normality. Initially, seven categories were formed: 1. school, leisure time and friends; 2. family life, including pets; 3. awareness of sibling’s death—when rather than if; 4. connectedness to many feelings and thoughts; 5. knowledge about brother’s or sister’s disease; 6. not being seen and feeling unacknowledged; and 7. losing part of oneself and one’s life. However, after discussions with all investigators, consensus decisions were made to rearrange some of the codes and to collapse two of the categories based on their connection and relationship; these were “5. Knowledge about brother’s or sister’s disease” and “6. Not being seen and feeling unacknowledged”. Many of the codes that had been placed in these two categories were then merged into either a category describing being part of a special family or a category describing school as a place for learning and friends. Ultimately, this resulted in five categories.

## 3. Results

Participating siblings, aged 6 to 14 years, all belonged to families with a child with palliative care needs due to a rare disease or sequelae of a trauma. Two sibling pairs participated, each pair belonging to a different family. All families were nuclear families and the ill children had been in need of palliative care for a year or more. The five categories were: being part of a special family; school—a place for leisure, friends, and learning; relentless feelings of guilt and self-blame; losses and separations; and awareness of death—not if, but when.

### 3.1. Being Part of a Special Family

Family, including grandparents and sometimes pets, meant a lot to all the siblings. They described their mother, father, brothers, and sisters with love. Some of the siblings who had a brother or sister with a rare disease mentioned that they were part of a happy family. Their particular circumstance had added another dimension to their life and the siblings reported perceiving themselves as having a positive attitude or feeling towards life—more so than their peers. This positive attitude was grounded in their family and usually passed on from their mother. The siblings often described themselves as happy, joyful, and funny. Almost all siblings reported being eager to learn, both in general and also more specifically about philosophical views on life. One of the siblings told us that they wanted to learn more about their ill sibling’s illness. This child had never seen their ill sibling healthy.

Grandparents played a big role in the siblings’ lives, since they felt that their grandparents always saw and acknowledged them. A feeling of being invisible was expressed by several of the siblings, most often related to situations at home.

All siblings described themselves as having a broad spectrum of feelings and emotions, ranging from anger to joy and happiness. They reported that they could express their feelings openly, both at home and with friends. Anger was often related to events happening within the family and directed towards their parents or healthy brothers or sisters. No sibling expressed any anger towards their ill brother or sister.

*“And then, I don’t get angry very often at home, but when I do, I get very angry and annoyed too.”*

*“Sometimes you can be annoyed with … or kind of irritated with your parents or your friends or when something is unfair.”*

The siblings mentioned medication as something that was essential for their ill brother or sister. They hoped for new, better drugs for them, especially if the ill sibling had a progressive disease. Siblings said that having a severely ill brother or sister made their family special. This was described in a positive way—it had given them a different perspective on life and meant that they found life precious. This was often stated in particular by siblings of children with rare diseases.

*“That, like that …. yeah, but that we are maybe not a normal family, that … because not all families are alike, but there are some that are a bit more unusual.”*

*“Yeah, I think that I can be, actually, because it is a … like, it’s my everyday life, but at the same time, it’s a much harder everyday life for me, and then you should be … and then you learn, you quickly learn to be happy and satisfied with what you have.”*

*“Well, our entire family is made up of happy, positive people.”*

### 3.2. School—A Place for Leisure, Friends, and Learning

The siblings highlighted the value of school, both as a place for learning but also for leisure and friends. They emphasized the importance of education and several siblings had ambitious goals for their future working life, such as becoming an author, a teacher, or a physician. Siblings also mentioned that their parents helped and supported them in their schoolwork. School gave them a break from illness and time with friends. Friends helped to ease their sadness, as it could be shared with them. One sibling mentioned that they were comforted by hugs from friends, and how much they liked being hugged when they were sad. At school, some siblings had not revealed that they had a severely ill brother or sister, which made them feel ordinary. One sibling, who had recently changed school, said that they longed for the fact that they had a brother or sister with a severe illness to be revealed so that they could be themself. Not being known as “the one” with a severely ill brother/sister could feel awkward. This illustrates the value of being a “special” sibling, as well as losing part of oneself (see first quote below).

*“When they knew, it felt good.”*

*“Because I’ve always dreamed of becoming a writer, so I’ve started writing stories and that kind of thing a lot in school. And I… and always when we get … and I … think I’m good at writing because every time there is a test in writing, I always have … I’ve always got everything right.”*

*“I see a child and then I kind of see a school and then I think that school is quite important, because you learn things then, yeah, you grow up and then you become something, or yeah, you start working, and then it’s kind of good that you have an education.”*

### 3.3. Relentless Feelings of Guilt and Self-Blame

Feelings of guilt and self-blame were described by siblings of children who had palliative care needs as the result of an accident. These siblings described in detail what had happened to their brother or sister and their role in it. One sibling was themself physically affected for a long time. An effect of feeling guilt and self-blame was crying, although not always out loud—described as a silent cry on the inside, without anyone else knowing.

*“You always feel a bit of guilt about what happened, but that’s just life. Instead of just waiting, you have to … instead of just thinking that ‘What were you doing? Why didn’t you do that?’ or ‘Why didn’t you realize that someone was missing?’ or ‘Why, why, why?’ you think in your head, but if I think like that all the time … I mean, nothing happens. You can never change it back, so it’s better to just leave it be.”*

**Figure 4:**
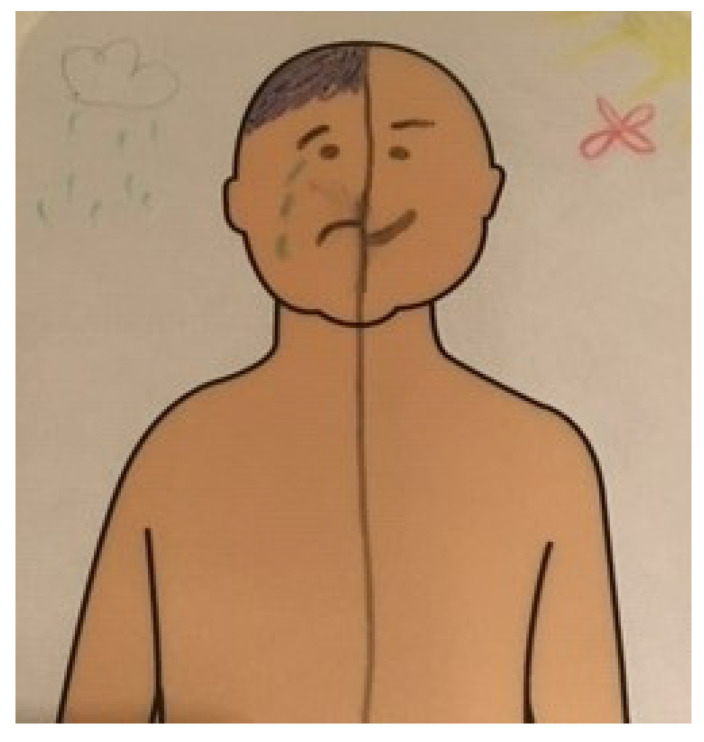
Relentless Feelings of Guilt and Self-Blame.

*“Yes, I don’t feel like… I don’t have the same feeling all the time, so… I’ll draw onehalf of the body happy and the other half sad Figure 4.”*

### 3.4. Losses and Separations

The siblings talked about losses and separations; the loss of the healthy brother or sister they once had, separation from friends and family, and the death of loved ones are all included in the category of losses and separations. Loss is so much more than just death. For example, losing the opportunity to be the one who can teach a brother or sister and be their role model was described as a great loss by some of the siblings. They referred to this as losing a part of themselves, a loss of normality. In the past, they could play with their brother or sister—now they could not. Separation, both direct and indirect, was also mentioned. One sibling expressed concern about his/her mother and father divorcing, though most losses were related to friends and friendships. Some children had lost friends because of their new circumstances of having a brother or sister with a severe illness; their families could not travel as much as they used to or live abroad. This was particularly true for siblings of children who had been injured in accidents and now had palliative care needs. The siblings experienced a loss of normality, but found a new normal.

Siblings of children affected by accidents also expressed an awareness that things that change family dynamics can happen at any time. One sibling expressed a wish for normality, without illness, and that they wanted to live “a normal life.” Access to “24/7 support” at home was vital for the ill child, the sibling, and the family as a whole; when such support was suddenly withdrawn it changed life for the family once again.

*“So, in one way you lose your regular life. But now this has become my regular life, so in one way I haven’t lost it. They’ve taken away our support, so we can’t do things.”*

*“No, when we were little or… I was three when he was born, so I remember that he… we could sit and play ball and stuff before he … like lost everything, so he could sit and so.”*

*“Yeah, like talking to him and so. You can’t really do that now, because you don’t know if he hears or understands. Yeah, and then to have someone to play with and so. That would have been fun too.”*

### 3.5. Awareness of Death—Not If, but When

Death was described as natural, although thinking about it made the siblings sad. Siblings of children with rare diseases told us that their brother or sister would die soon. Some said it should have happened already. This topic came naturally to the siblings, and it was not a matter of whether the brother or sister would die, but when. One sibling opened our meeting by saying: *“Yeah, I’ve been thinking a lot about what will happen when X has died, will we be happy again? Will we ever be able to laugh? Yeah, I’ve been thinking a lot about it.”* Siblings of children with palliative care needs due to an accident did not mention death. Their worries were connected to living with uncertainty—that anything could happen at any time, which could change life entirely.

*“You don’t really know what is going to happen either. Yeah, maybe a bit helpless, because there is no real antidote or anything like that yet. I don’t know, I think there’s research being done on this, but they’ve not come up with that much. So it will be difficult to help X, but it may be possible to help others. So it’s like that, a bit helpless.”*

*“I don’t know, but I think I learned a lot from my mum, because in the beginning when I was younger I didn’t really understand that my big sister was ill. But then I’ve lived with the fact that she’s become more and more ill and just from that I think I’ve learned a lot, but then mum and dad have talked to me a lot about it and then I’ve also learned a lot. I think that’s also why I’m a positive person.”*

## 4. Discussion

Using the four different communication tools, siblings presented stories about what is important in their lives when living with a brother or sister with palliative care needs. Observations from this study are novel. To our knowledge, this is the first study to report that siblings of children with rare diseases expect the death of their brother or sister while at the same time reporting that they are part of a happy, special family. These siblings felt that they viewed life as more precious than their peers. In contrast, siblings of children with palliative care needs due to accidents described relentless feelings of self-blame and guilt. Our observations were limited due to the number of siblings participating, but their narratives differed depending on the cause of the ill brother’s or sister’s palliative care needs. Overall, the siblings described having active lives, with friends and activities, but had an internal sense of insecurity, knowing that life could change at any time. This reflects the human ability to adjust to certain situations—in this case as a young sibling and part of a larger system, the family.

The siblings were aware that their brother or sister could die at any time, especially if the brother or sister had a rare disease. This seemed not to be age-dependent among the siblings we spoke with, who were 6–14 years of age. Younger children may understand the permanence of death without truly realizing that it is forever, something that older children are more likely to comprehend [20]. Our findings are in line with those of Gaab and co-workers who, based on interviews with bereaved siblings whose brother or sister had palliative care needs before dying, reported that death is a companion to these siblings. Most of them wanted to be informed about the imminent death, to be able to be involved. This is something that we have also seen in our previous work among siblings of children with cancer, although death had not been talked about in this way [10]. It may be that, because cancer is often curable, there is more hope among those affected than for many siblings of children with rare diseases or sequelae of trauma, where the hopes of cure or improvement are limited. The study by Gaab, Owens, and MacLeod [21] was not able to identify the difference that we observed in the current study. Jaaniste and co-workers recently reported that siblings never or rarely talk about a severely ill brother’s or sister’s illness or death [9]. Not surprisingly, siblings’ satisfaction with family communication was significantly associated with family cohesion.

The impact of the severe illness on the family was considerable; however, the siblings stated that they were part of happy families. This happiness and positive view of life was particularly emphasized by those whose brother or sister had a rare disease. Siblings of children who had suffered an accident did not express such joy or happiness. They spoke instead about uncertainty and living with the knowledge that everything could change in an instant. Losses and separation were also mentioned more often by these siblings. This could be in reference to personal losses or losses for the entire family that implicitly affected the sibling’s life. Most of the losses were not related to death; the siblings instead referred to the loss of friends or the normal life they once had. Much of the above, i.e., the impact of illness on the family, uncertainty, losses, and separations, has been reported by parents, but it is rare to hear this from young siblings [22].

The difference in the experiences and feelings described by this small sample of siblings in relation to the cause of their brother’s or sisters’ palliative care needs was novel. We have not previously encountered this in either clinical work or research. The rapid change from a normal life to a life with constant uncertainty may explain the feelings and concerns expressed by siblings of a child with palliative care needs due to trauma. Living with such uncertainty and self-blame at a young age may have a long-term impact on their development and wellbeing. Further knowledge is needed to find ways for healthcare professionals and social systems to support these children and help them cope with such feelings.

The healthcare and social systems around the ill child and family should not be taken for granted. All these families had been given the chance to receive respite care at the hospice. Respite care has been shown to be of value for the whole family [4,23]. However, the siblings in our study did not talk much about the “good side” of it. Instead, they emphasized that rapid changes in the healthcare services provided could impact the whole family in a negative way. The family, as a system, is vulnerable to changes [24].

Communication tools are used worldwide in pediatric cancer care to help affected children find ways to express themselves and have their voice heard [14,25,26,27]. This led us to believe that siblings of a brother or sister with palliative care needs due to other causes than cancer might also benefit from using communication tools to express their experiences and feelings. A strength in this study was the use of four different tools in the conversations, since some siblings preferred letters and words rather than pictures and vice versa. We believe the variety of tools helped the siblings, some as young as six years old, express what was important to them.

A limitation of this study was the small and homogeneous sample of only Swedish-speaking siblings, and that the participants were mostly girls. Given the small sample and the wide age range (6–14 years), it is not possible to study to what extent additional factors, such as age, gender, deaths within the family, bullying, coping style, and health status, may impact the siblings’ daily lives. Nor can our findings relating to the brother’s or sister’s cause of palliative care needs, i.e., being part of a special happy family or being aware of one’s brother or sister’s imminent death, be explained. Larger studies need to be performed to confirm such findings. Some might also consider the use of a selection of communication tools that lack a theory to be a limitation, although the tools that were used have their origin in both clinical work and research. In addition, conventional content analysis was used to explore the siblings’ narratives because of the lack of theory, as is recommended. The presence of parents during two of the interviews could be viewed as both a strength and a limitation. A parent’s presence can be a source of security but may also hinder children in being able to express themselves [28]. In one case, the parent’s presence helped the child feel secure and able to talk freely; the sibling in this case was a seven-year-old boy. Although he did not thank his parent verbally, his body language indicated that his parent’s presence made him feel secure. In the other case, the sibling was a 14-year-old girl, and, in contrast, the parent exploited the conversation, and it became obvious that parent and daughter viewed things differently.

In this study, we noted that siblings living with children with rare diseases seemed to have internalized thoughts that their brother or sister would die. Further, they felt that they belonged to a special and happy family. Siblings of children with palliative care needs due to an accident expressed relentless feelings of self-blame and guilt. Communication tools, such as those used in this study, may be useful for healthcare professionals to initiate communication and further understand and support each sibling’s needs. Healthcare professionals may also introduce some of the tools to parents and teach them how to use them, for example Bear cards, since they may help open up family communication and give siblings a voice for the undisclosed feelings they carry, thereby reducing the risk for poor long-term health outcomes for siblings.

## Figures and Tables

**Table 1:** Siblings included in the study.

Number	Age of the Sibling (Years)	Sex of the Sibling	The Brother’s or Sister’s Illness/Disability
1	10	Female	Rare disease
2	6	Female	Brain injury due to trauma
3	8	Female	Brain injury due to trauma
4	10	Female	Brain injury due to trauma
5	14	Female	Rare disease
6	14	Male	Rare disease
7	8	Female	Rare disease
8	7	Male	Rare disease
9	14	Female	Rare disease

## Data Availability

Data cannot be obtained because they contain personal information.
